# Polycystic Ovarian Syndrome roots needs to be rooted out at the outset: Will early screening help?

**DOI:** 10.12669/pjms.37.5.4509

**Published:** 2021

**Authors:** Sikandar Hayat Khan, Uzma Urooj

**Affiliations:** 1 Dr. Sikandar Hayat Khan, FCPS Chemical Pathology, PgD Endocrinology & Diabetes and MSc Cancer, Molecular Pathology and Genomics, Head Department of Pathology, Naval Hospital Islamabad, Islamabad, Pakistan; 2 Dr. Uzma Urooj Consultant Gynecologist, PNS HAFEEZ Hospital, Islamabad, Pakistan

Evolving through time, mankind adopted various lifestyles to comfort themselves which were completely different from the life lived by our ancestors. Breeding over this evolutionary “comfort zone” are the curses involving extremely processed food with much more controlled local surroundings owing everything to the contemporary technological boom.^1^ While we are seeing “*Homo sapiens*” exploring the ins and outs of our milky way, still the curses of new form are pinching the smoothness of life at home. Contrary to the ease and exuberance our species enjoyed, the “Human Metabolism” have seen some of the worst hits in the form of metabolic syndrome, Type-2 Diabetes, Hypertension and list of Cardio Vascular Disease. Emerged in between these metabolic disorders also includes “Polycystic Ovarian Syndrome (PCOS)”, which follow almost similar genetic footprints originating from life style influences acquired over the course of human evolution.^2^

The disease at the onset may have lifestyle epigenetic triggers which overtime with poor lifestyle management may lead to development of PCOS phenotypes. Clearly racial, ethnic and psychosocial differences allowed various PCOS presentation, which slightly differ in clinical features. Lacking a general consensus on defining a common criteria for PCOS, several definitions have emerged from this scientific world like Rotterdam criteria.^3^ The unifying factors for most of these criteria is most often than not the establishment of ‘hyperandrogenemia’, which is not just having a temporal and spatial aspect including contributors such as age, race specific visceral obesity, regional variations in hair growth.^4^ Underlying variable presentations of PCOS, there are usually cysts in ovaries which disallow the functionality of reproductive cycles.^4^ However, over time these little alteration can lead to complications like infertility, facial scaring and metabolic association like type-2 diabetes, hypertension and higher frequencies of Cardio Vascular Diseases (CVD).^5^ Relationship of these physical and metabolic manifestations in youth have earned this disorder a name in common parlance i.e., “Thief of womanhood”.

Undoubtedly, the seeds are sown early in life where the ignorance of exuberance and deception of luxury leads future stage setting for this disease.^6^ Unstoppable these pathogenic pathways can lead to an ever increasing modification in ovarian tissues and hormones, which could compromise female’s very delicate reproductive cycle. These issues not only distraught facial appearance but in low socioeconomic groups can lead to a social dogma.^7^ The issue never ends here as the disease needs not only demands cosmetic treatment but also management of reproductive issues and infertility in the earlier stages of life. The economic impact of the disease management can in no way be undermined in poor countries where the same resource can be diverted to manage some of the major planetary killers including cancers, cardiovascular diseases and diseases related to lack of primary healthcare facilities.

Acknowledging and appreciating the issue will require timely data as some of the regional data clearly points towards early onset in certain regions like South Eastern societies.^8^,^9^ While treatment after diagnosis will not remain sub optimal and prolonged, but definitely expensive and tagged with social dogma in most set ups.^7^ While a lot has been worked up in terms pathogenesis, diagnostic criteria, hormonal diagnostics and radiological diagnosis still it is felt that there is clear and manageable neglect in being adopting a proactive approach towards this multi-faceted disease by not adopting primary prevention in earlier years of life. The concept of primary prevention becomes more central once we realize that the available diagnostics are not providing specific, with available criteria remain overlapping and not racially tailor made and therapeutics least curative. Provided the white wash cosmetics is being done to hide underlying volcanic seismic metabolic activity, nonetheless the patients become destined to get late onset atherosclerotic cardiovascular diseases (ASCVD) along with higher prevalence of cancer and metabolic syndrome.^5^ So addressing the metabolic illness as early as possible seems the most plausible and cost effective proposition.

So why not early screening? The interventions in young girls may start early by adopting simplistic and basic measures in a cost effective manner. BMI and waist to hip measures and including physical activity and eating habits in syllabi could be few preliminary steps which must be given due consideration followed by adolescent females being taught specifically about the disorder and ways to deal with it as measure of primary prevention. In conclusion it can be suggested that an early screening from a well collected history, screening program in schools and colleges and media coverage can reduce the futurist impact of this potential threat at the outset. Healthcare and educational policy makers must be included in such policy making.

## Author’s Contribution:

**SHK**: Idea conception, Review of data, manuscript writing, referencing.

**UU:** Manuscript writing, data review and finalization. Both authors approved manuscript’s final version.
